# Circulating Histones to Detect and Monitor the Progression of Cancer

**DOI:** 10.3390/ijms24020942

**Published:** 2023-01-04

**Authors:** Desislava K. Tsoneva, Martin N. Ivanov, Nikolay Vladimirov Conev, Rostislav Manev, Dragomir Svetozarov Stoyanov, Manlio Vinciguerra

**Affiliations:** 1 Department of Medical Genetics, Faculty of Medicine, Medical University of Varna, 9000 Varna, Bulgaria; 2 Department of Stem Cell Biology and Transplantology, Research Institute, Medical University of Varna, 9000 Varna, Bulgaria; 3 Department of Anatomy and Cell Biology, Research Institute, Medical University of Varna, 9000 Varna, Bulgaria; 4 Clinic of Medical Oncology, UMHAT “St. Marina”, 1 “Hristo Smirnenski” Blvd., 9000 Varna, Bulgaria; 5 Department of Propedeutics of Internal Diseases, Medical University of Varna, 9000 Varna, Bulgaria

**Keywords:** liquid biopsy, histones, cell-free DNA, cancer

## Abstract

Liquid biopsies have emerged as a minimally invasive cancer detection and monitoring method, which could identify cancer-related alterations in nucleosome or histone levels and modifications in blood, saliva, and urine. Histones, the core component of the nucleosome, are essential for chromatin compaction and gene expression modulation. Increasing evidence suggests that circulating histones and histone complexes, originating from cell death or immune cell activation, could act as promising biomarkers for cancer detection and management. In this review, we provide an overview of circulating histones as a powerful liquid biopsy approach and methods for their detection. We highlight current knowledge on circulating histones in hematologic malignancies and solid cancer, with a focus on their role in cancer dissemination, monitoring, and tumorigenesis. Last, we describe recently developed strategies to identify cancer tissue-of-origin in blood plasma based on nucleosome positioning, inferred from nucleosomal DNA fragmentation footprint, which is independent of the genetic landscape.

## 1. Introduction

### 1.1. Liquid Biopsy

**Figure 1:**
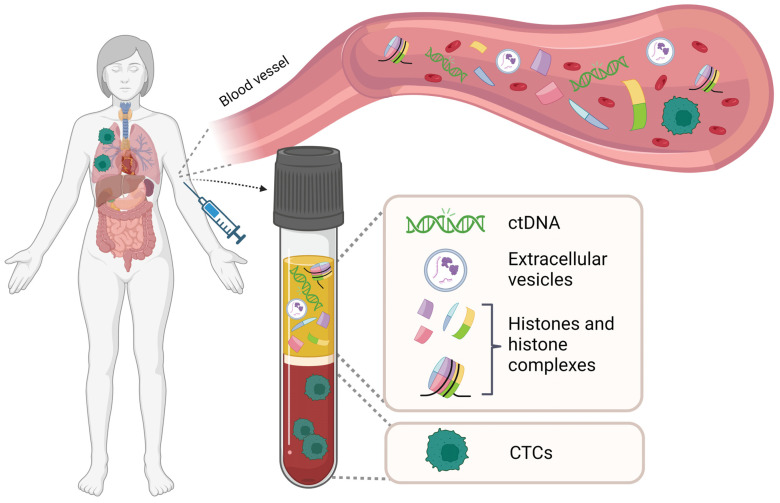
Schematic illustration of the components in a liquid biopsy. Liquid biopsy obtained from peripheral blood contains different tumor-associated materials such as circulating tumor cells (CTCs), circulating tumor DNA (cfDNA), extracellular vesicles (EVs), free histones, and histone complexes. Such fractions can be isolated and analyzed for tumor-specific aberrations at the genomic, transcriptomic, proteomic, and metabolomic levels. Figure created with BioRender.com.

Liquid biopsy represents a minimally invasive, convenient, and cost-effective method for molecular diagnosis. It is increasingly recognized that liquid biopsy can provide comprehensive information on the molecular landscapes of cancer. Methods for isolation and analysis of liquid biopsies have rapidly improved over the past few years, providing greater insights into tumor characteristics such as progression, staging, heterogeneity, gene mutations and clonal evolution. Although the technology of liquid biopsies is still developing, its non-invasive nature promises to open new scenarios in clinical oncology. Liquid biopsies include circulating tumor DNA (ctDNA), circulating tumor cells (CTC), tumor extracellular vesicles (TEV), and circulating histones and histone complexes (Figure 1).

Circulating tumor DNA liquid biopsy has been studied most extensively in patients with established metastatic disease and has been applied in some routine clinical applications using next-generation sequencing (NGS) technologies [1,2]. Very often, tumors are characterized by specific genetic mutations: ctDNA assays revolutionized the field, being able to routinely assess somatic alterations of interest (point mutations, chromosomal aberrations, epigenetic modifications, and DNA fragmentation size), moving the monitoring approach from tumor tissue-based to blood-based testing. Tumor alterations detected through routine tumor tissue analysis are detected in ctDNA with a sensitivity of ~80–90%, depending on disease location and tumor burden, displaying a robust correlation with overall ctDNA signature [1,3]. CtDNA-based liquid biopsy can be employed for primary cancer screening. Most cancer types are much more likely to be cured when diagnosed early. In this respect, five tests have been approved by the FDA so far, which identify point mutations in cancer-related genes such as KRAS, EGFR, PIK3CA, tumor mutation burden, microsatellite instability, insertions and deletions, and methylation patterns [4,5]. In the frame of malignant disease monitoring and progression, ctDNA has also been used as a marker for guiding therapy and minimal residual disease (MRD). The latter is used for the detection of residual disease, based on the presence of cancer-derived molecular biomarkers, when the bulk cancer is not detectable by conventional investigations, such as medical imaging, for instance, in melanoma and colorectal carcinoma (CRC) [6,7,8,9,10,11]. Unfortunately, ctDNA in the bloodstream has a very short half-life, ranging from 30 min to 2 h, causing problems in several areas, including tracking tumor heterogeneity, precision treatment, and rigid protocol standardization (Table 1).

Whereas ctDNA can only be analyzed at the genomic level, CTC can be dissected at transcriptomic, genomic, and proteomic levels either in bulk or as single cells. CTCs originate from the primary tumor and are believed to be directly involved in the metastatic cascade. CTCs are disseminated from the primary tumor, surviving treatment or surgery in the systemic circulation, and start tumor formation at a site distant from the primary tumor. During the metastatic process, cancer cells undergo the epithelial-mesenchymal transition (EMT), acquiring the migratory and invasive ability to invade the surrounding stroma. They then intravasate and survive in the vasculature as CTCs, eventually extravasating at distant organs and giving rise to metastatic tumors [12]. During EMT, the expression of epithelial markers such as E-cadherin, EpCAM, and cytokeratins is lost, and the expression of mesenchymal markers such as N-cadherin, vimentin, and fibronectin is increased. Flow-cytometer-based detection of the above-mentioned markers is a way to identify CTCs and investigate their aggressive behavior [13]. EpCAM-based methods have been previously considered the gold standard for CTCs detection [14,15,16]. Nevertheless, tumors could be characterized by low EpCAM expression or be EpCAM-negative, as in the case of malignant melanoma and medulloblastoma [17,18,19,20,21,22,23,24]. Furthermore, due to the lost expression of EpCAM during EMT, conventional EpCAM-based methods could be inefficient in capturing CTCs [25,26,27]. More sensitive biomarker-independent CTC isolation techniques have been recently developed with surface-charged superparamagnetic nanoprobes capable of different EMT subpopulation CTC capture from a tiny volume of blood [28,29]. Moreover, the development of sophisticated single-cell analysis technologies has allowed the dissection of heterogeneity within CTCs [29]. CTC from liquid biopsies can provide non-invasive diagnostic and therapeutic information for several cancer types. Moreover, CTCs isolated from CRC patients harbored KRAS mutations, which are a strong predictor ofresistance to EGFR inhibitors [30]. Nevertheless, critical issues remain in CTC detection sensitivity, technical reproducibility, and identification accuracy [29] (Table 1).

Extracellular vesicles (EVs), also referred to as membrane vesicles, have a subcellular structure with a lipid bilayer similar to a cell membrane and are classified as exosomes and microvesicles. Tumor EVs (TEVs) are EVs produced by tumor cells and mediate multiple biological cancer processes, including cell growth, proliferation, and migration, through their cargos transferred between different cells [31,32]. TEV alterations before and after therapy also show great potential for therapeutic response monitoring [33,34]. TEV carry the RNA transcriptome, proteins, lipids, and DNA, making them small packages of multi-analyte biomarkers [35]. For instance, various studies have suggested that the cargo contained in TEV, such as the microRNAs released from cancer cells, can modulate tumor growth, invasion, metastasis, angiogenesis, and drug resistance, thus showing great potential as therapeutic targets [36,37]. TEVs are intensely researched for screening and disease monitoring in cancer of the skin, lung, prostate, and breast, glioblastoma, lymphoma, and head and neck squamous cell carcinoma [38]. Critical to TEVs utilization as biomarkers are the isolation/separation and purification techniques, especially due to their heterogeneity. While not limited to those methods, TEVs are often isolated by ultracentrifugation, which eliminates several types of contaminants, including large serum proteins, or by density gradient, size exclusion chromatography, magnetic bead immunoaffinity, or lipidomics/mass spectrometry [39]. Absolute purification is currently an unrealistic aim. However, widely accepted guidelines for distinct approaches and the corresponding controls have been defined and continue to be updated on a regular basis [40,41]. Similarly, comparative studies on techniques of isolation and analysis of CTCs and ctDNA and their preanalytical variables affecting the detection quality are increasing, providing insight into standardized procedures and their potential improvement [42,43,44]. A list of long-established and novel techniques for ctDNA, CTCs, and EVs detection is provided in Table 1 [45,46,47,48].

Detection of cancer-related variations in nucleosome or histone levels/post-translational marks (PTMs) in biological fluids such as plasma and serum could also serve as useful biomarkers in cancer detection, diagnosis, and management [49]. Such biomarkers offer many advantages. Histone proteins have a long half-life and are very stable in the blood. In terms of the diagnostic value of liquid biopsies, anti-nucleosome antibodies have been shown to be >2 fold more sensitive compared to anti-DNA antibodies in the detection of autoimmune diseases [50]. Historically, the potential diagnostic value of intact nucleosomes or histones has been considered limited since the diseases associated with accelerated cell death, such as cancer, are also associated with an elevated level of circulating cell-free nucleosomes/histones [51]. The purpose of this review is to provide a comprehensive overview of circulating histones as liquid biopsies and their application for the detection, prognosis, and monitoring of cancer treatment outcomes (Table 1).

### 1.2. Circulating Histones

In eukaryotic cells, DNA is compacted into chromatin by wrapping around histone complexes called nucleosomes [52]. The nucleosome is composed of approximately 147 base pairs of DNA coiled around two H2A-H2B dimers and one H3-H4 tetramer [53,54]. Adjacent nucleosomes are connected through a “linker DNA” of approximately 20–80 base pairs in length, bound by histone H1 [52,53]. The nucleosomes are the core of chromatin and are essential for consistent and accurate DNA replication, transcription, and repair. It is clear that the correct orchestration of nucleosome regulation is vital for genome integrity.

The numerous protein-protein and protein-DNA interactions make the nucleosome highly stable [53,54], yet not static [55]. The histones contain so-called N-terminal histone tails, which protrude out of the octamer and are subjected to PTMs, which alter the conformation and the interaction properties of the nucleosome. Furthermore, nucleosomes consist of distinct histone variants, resulting in structural variations linked to distinct functions. The nucleosome positioning is also very dynamic. As the level of chromatin compaction lies at the center of gene expression regulation, the nucleosomes should be able to alternate their place and state. To do so, the nucleosomal histones interact with various proteins in a highly organized spatiotemporal manner. Furthermore, to maintain genomic stability, nucleosomes are continuously undergoing assembly and disassembly [56]. Both processes are directly linked to histone synthesis and degradation [56]. Histone proteins have a long half-life of approximately 220 days [57,58,59]. Nevertheless, it should be taken into account that histones in different cells/tissues and chromatin regions exhibit significant differences in their half-lives [60]. For instance, the histone turnover in hepatocytes was shown to occur relatively fast (18–61 h) compared to the brain (~72 days) [59]; however, this was notably slower in comparison to fast turnover proteins (~9–11 h).

Circulating histones and nucleosomes can be detected in healthy and diseased conditions among individuals. While the main source of circulating histones is believed to be apoptotic and necrotic cells [61], histones can be secreted into the extracellular space by activated cells, acting as damage-associated molecular pattern molecules [62,63,64,65]. Neutrophils can exert a specific immune defense mechanism, referred to as “neutrophil extracellular traps” (NETs) [66], in which genomic DNA, core histones, and antimicrobial factors are released by neutrophils to degrade invading pathogens [66,67]. Of note, such extracellular traps have also been reported in subsequent studies for macrophages, mast cells, and eosinophils [68,69,70,71,72,73], further supporting the role of histones in the immune response [62,63,64]. NETs can subsequently cause a specific form of cell death (“NETosis”), leading to further histone release [74]. Furthermore, histones released in the extracellular space in response to apoptotic signals can trigger an apoptotic cascade [75]. For example, hyperacetylated H3.3 accumulates in the extracellular space due to resistance to proteasomal degradation and facilitates apoptosis in lung cells, resulting in H3.3-mediated lung injury [75]. Administration of histone antibodies resulted in reversed cytotoxicity [75,76,77], directly linking histone release as a driver of toxicity.

Together, previous findings highlight the potential significance of circulating histones in the modulation of inflammation, which is tightly linked to cancer pathogenesis [78].

#### Methods of Detection

For the purpose of this review, we will discuss three main techniques applicable to the detection of circulating histones: enzyme-linked immunosorbent assay (ELISA), proteomics, and ImageStream (Table 2).

ELISA is an easy-to-use and access antibody-based technique that allows for the quantitative measurement of several proteins in lysates or bodily fluids. ELISA is moderately expensive, allowing for multiple measurements and assay standardization. In fact, ELISA is often used to detect selected cancer-associated PTMs on nucleosomal DNA [79], such as histone H3 lysine 4 trimethylation (H3K4me3) and H3K27me3 [80,81,82,83]. ELISA-mediated detection of four circulating nucleosome-associated markers (H2AK119Ub, H3K9Ac, H3K27Ac, and the total level of nucleosomes) achieved sensitivity scores (75% and 86%) for stage I and II colorectal cancer, respectively [84]. Similarly, changes in circulating nucleosomes on the epigenetic and structural levels, measured by ELISA, detected pancreatic cancer with a higher specificity compared to carbohydrate antigen 19-9 (CA 19-9) [85]. Combining circulating nucleosomal analysis with CA 19-9 resulted in further increased sensitivity and specificity of pancreatic cancer detection [85]. These results indicate the ability of ELISA to serve as a reliable approach to cancer screening. Furthermore, recent studies developed ELISA-based methods for NETs detection by measuring the levels of citrullinated histones H3 (H3Cit) [86,87]. Interestingly, high levels of circulating H3Cit, measured by ELISA, were found specific to cancer patients, as hospitalized and severely ill non-cancer patients and healthy individuals did not show elevated H3Cit [88]. Notwithstanding, ELISA-based methods have shown low reproducibility and significant error ranges [89] (Table 2).

Immunoassays have proven valuable and reliable for the quantification of circulating histones/nucleosomes or specific modifications on histones in fluid samples. However, when the interest is the identification and quantification of unknown cancer-relevant histone PTMs, immunoassays lack such unbiasedness. Instead, recent research defines novel proteomics analyses as a powerful approach to biomarker discovery (Table 2). The use of mass spectrometry and multiple reaction monitoring was able to quantitatively detect the concentration of circulating histones and establish the circulating histone levels as biomarkers for septic shock diagnostics and as predictors of patient prognosis [90]. Similarly, immunoprecipitation followed by liquid chromatography and tandem mass spectrometry discovered augmented H3.1-positive circulating nucleosomes in CRC patient samples, and a panel of 13 PTMs on circulating histones, three of which reflected the epigenetic profile of CRC tumor tissues [91]. Additionally, high levels of H3.1-positive nucleosome, H3, H4, and H2A1 were able to detect CRC, irrespective of PTMs [91]. Recently, Fedyuk et al. developed a novel single-molecule imaging approach, which could establish the epigenetic profile of plasma-isolated nucleosomes, DNA methylation, and expression of cancer-specific protein biomarkers at high resolution. Application of the approach achieved high accuracy in detecting colorectal and pancreatic cancer, including early disease stages. In fact, integration of all measurements (protein biomarkers, DNA methylation, and histone PTMs) through machine learning resulted in 92% sensitivity, 85% specificity, and 92% precision, which is superior to predictive models, relying on either of the measurements alone [92].

ImageStream is a multi-channel imaging technology that combines phenotypic sensitivity, the multiplex properties of flow cytometry, and the visual power of microscopy to provide extensive information on a particle or a cell of interest [93]. Recent studies have shown the promising results of applying ImageStream to profile histone signatures in lean metabolic associated fatty liver disease (MAFLD) and non-alcoholic fatty liver disease (NAFLD) patients. Interestingly, while nucleosomes were poorly associated with MAFLD, the levels of circulating histones macroH2A1.1 and macroH2A1.2 were significantly reduced in lean MAFLD patients. Furthermore, histone signature, specifically macroH2A1.2, H2B, and H4, was found to reflect the severity of MAFLD [94]. In the context of pediatric NAFLD, macroH2A1.2 showed the reverse expression change, with significantly increased circulating levels in NAFLD children, compared to healthy controls [95]. Taken together, we could hypothesize that (1) circulating histones could act as promising biomarkers, detecting onset and disease progression and (2) ImageStream technology is a robust method to evaluate small peptides such as histones in a limited amount of sample. Importantly, ImageStream was able to detect microparticles in complex fluids, including whole blood, platelet-rich, platelet-poor plasma, and leukocyte supernatants [93]. With this approach, time-consuming preparation is not essential, providing an opportunity for procedure standardization.

The main advantage of ImageStream is the ability to detect multiple biomarkers, including histones and cancer cells, in a low amount of sample at a relatively low cost (Table 2). Integration of multiple parameters has proven valuable in increasing sensitivity, identifying associations, and determining prediction scores [96] that may advance patient anti-cancer treatment decision-making.

The main disadvantage of ImageStream could rely on inter-observer variability. Data analysis of ImageStream-produced information-rich image sets is often performed in a manual and error-prone manner, using a fraction of the object features. Therefore, the reproducibility of the results is directly linked to the level of experience of the analyst (Table 2). However, open-source software such as Image Data Exploration and Analysis Software (IDEAS), which is coupled to ImageStream for image analysis and statistics, often provides options for machine learning.

In recent years, increasing evidence indicates the prognostic power of artificial intelligence (AI) in disease detection and monitoring. AI can process complex data, including image data, and extract relevant information. For instance, AI was applied to CT imaging data from 300 head and neck cancer patients, aiming at predicting locoregional recurrences (LR), distant metastases (DM), and overall survival (OS). Notably, the developed AI model was able to anticipate the same outcome as when CT imaging, PET imaging, and clinical variables were utilized together in the analysis. Furthermore, combining the two methods achieved an enhanced predictive score compared to either model alone, demonstrating the ability of AI to recognize image patterns that traditional radiomics might miss [97]. Similarly, AI-mediated analysis of tumor-infiltrating lymphocytes in advanced non–small-cell lung cancer (NSCLC) identified three immune phenotypes that correlated with response to immune checkpoint inhibitors and progression-free survival, which is challenging by manual quantification [98].

Utilization of such models could serve as an adjacent biomarker that aids the decision of the most appropriate anti-cancer treatment plan. Nevertheless, AI implementation in clinical routines is currently lacking. However, the progress in AI in disease monitoring and therapy decision-making highly suggests the adaptation of such methods in the near future.

## 2. Tracing the Tissue of Origin of Circulating Histones

A crucial limitation of circulating histones and histone complexes as biomarkers for cancer detection and monitoring is the identification of the detected histones tissue-of-origin, which is of utmost importance for early detection and diagnostics of cancer, and disease progression monitoring. The main challenges lie in establishing the composition of circulating histone complexes and the prerequisite for genetic differences between healthy and tumor samples, which is further exacerbated as tumors often present no or unknown genetic mutations within the histones. A notable exception may be diffuse midline glioma (DMG), a highly morbid pediatric brain tumor: up to 80% of DMGs harbor mutations in histone H3-encoding genes, which is associated with poor prognosis [99,100]. Among these, recurrent and somatic H3.3K27M mutations can be detected using qPCR at the ctDNA level in DMG cerebrospinal fluid (CSF), plasma, and primary tumor specimens, using a standardized protocol that does not involve assessing the protein levels [100]. Moreover, as previously mentioned, H3Cit, which is involved in NETs formation [101], can be promptly measured by ELISA in biological fluids and correlates with the diagnosis and prognosis of several tumor types [86,88,102]. However, H3Cit is released from neutrophils and it is not tumor-specific.

Cell-free DNA (cfDNA) generally refers to DNA bound to histone complexes [103,104], as naked DNA in circulation is rapidly digested by nucleases [105,106,107]. Furthermore, previous analysis of circulating DNA fragments showed peaks corresponding to approximately 147bp [108]. Snyder et al. suggested that the fragmentation pattern of circulating DNA could reveal information on the epigenetic signature of the fragment-releasing cell [109]. Given that cells and tissues in healthy and diseased conditions are characterized by specific epigenetic profiles [110,111,112,113], sequencing and mapping of cfDNA fragments, and thus, nucleosome occupancy could identify the tissue-of-origin of the cfDNA. The authors showed that the cfDNA fragmentation pattern is indicative of nucleosome positioning at the transcriptional start site and gene bodies and correlates with gene expression signature, cell lineages, and tissue types [109]. Importantly, nucleosome spacing in cfDNA, isolated from patients with diverse solid cancer types, was able to identify various non-hematopoietic contributors. Of note, in some cases, the top-ranked cell lines and tissues analyzed were aligned with the identified patient’s cancer type, indicating a potential value of the approach in cancer diagnostics. In comparison, the top-ranked correlations in healthy individuals were lymphoid and myeloid lineages [109], which is in line with hematopoietic cell turnover as the prime cfDNA source [114]. These findings suggest that the measurement of circulating histones and histone complexes, together with the establishment of the nucleosome footprint by sequencing the associated DNA, could be applied to establish an elaborate cancer (location) detection and monitoring approach.

Nevertheless, Snyder et al. showed that in some instances, the highest-ranked cell lines were aligned poorly with the cell types, which could be associated with an underrepresentation of the specific cancer types in the used datasets [109]. Therefore, the potential application of nucleosome footprinting in cancer care could depend on advancements in tumor heterogeneity characterization and the generation of detailed referenced datasets. Furthermore, relatively low coverage of transcriptional start sites was achieved [109], which could render the approach less powerful. While, currently, ex vivo nucleosome footprinting seems unlikely to outperform the sensitivity and specificity of the currently applied diagnostic methods, it might prove valuable in characterizing cancers with an unknown primary origin, supplementing diagnostics and invasive biopsies-mediated cancer subtyping.

## 3. Circulating Histones in Hematological Malignancies: Markers, Predictors, and Therapeutic Potential

Hematologic malignancies originate from blood cells or blood-forming tissue and are subdivided depending on the type of the affected cell. Leukemia is a hematologic malignancy that develops when leukocytes are produced abnormally, causing high levels of not properly functional white blood cells. Leukocytes include neutrophils, eosinophils, basophils, monocytes, and lymphocytes, all of which were shown to release extracellular traps. Acute leukemia patients have shown increased levels of NETs biomarkers [115,116] compared to healthy individuals. These findings suggest that aberrantly developed leukocytes, similar to fully functional leukocytes, could release extracellular traps in the circulation. That brings into question whether the release of extracellular traps could be utilized as a defense mechanism by the organism or as a progression mechanism by leukemic cells. Early studies suggested that the administration of extracellular H1 histone caused cytotoxicity in 19 of 21 leukemia-derived cell lines and 11 of 16 patient-derived tumor samples without affecting bone marrow cells and peripheral blood mononuclear cells [117]. Furthermore, histone H1 was able to inhibit tumor growth when injected into Burkitt’s lymphoma mouse model [117]. The effects of purified histones and NETs, which are composed of histones, DNA, and granule proteins, likely differ, especially due to the high toxicity of the positively charged free histones. It should be noted that a high concentration of purified histone H1 was used −200 µg/mLin cell lines and patient-derived tumor samples and 250 µg/mLin the mouse model. Conversely, later studies showed that cell-free histones in plasma samples of leukemia patients stimulated the attachment of leukemic cells to endothelial cells by inducing the expression of endothelial adhesion molecules. Furthermore, the histone-mediated adhering of leukemic cells to endothelial cells resulted in increased survival of leukemic cells to spontaneous and chemotherapy-induced death, directly linking extracellular histones to leukemia progression [115,116].

Several studies have shown drastically increased circulating histones and histone complexes in patients with hematologic malignancies [115,116,118,119,120] (Table 3). Similarly, high levels of nucleosomes containing the histone H3.1 isoform (Nu.Q-H3.1) were found in non-Hodgkin lymphoma (NHL), acute myeloid leukemia (AML), and especially in acute lymphocytic leukemia (ALL), with a median of 276 ng/mL, 284 ng/mL and 585 ng/mL, respectively, as compared to 40 ng/mL for healthy individuals [119]. Furthermore, Mueller et al. indicated that circulating histone complexes could act as a predictive marker of chemotherapy response in acute myeloid leukemia patients [121]. Levels of circulating nucleosomes were also found to correlate with lymphoma progression and detect advanced (stage III and IV) lymphoma with 100% sensitivity [122], which was subsequently suggested to be mediated irrespective of apoptosis [123]. Nevertheless, studies with large patient cohorts and advanced detection methods are lacking. Therefore, it is currently unlikely that histone level measurement and therapies modulating histone levels in hematologic malignancies could enter the clinics in the near future.

## 4. Circulating Histones in Solid Cancers: Detection, Monitoring, and Tumorigenesis

Diagnosis of solid cancers often requires biopsy acquisition through invasive procedures, which are frequently accompanied by time-consuming analysis. Furthermore, due to their invasive nature, traditional biopsies do not allow for interval testing and therefore lack disease-monitoring abilities. A list of studies indicating measuring histones, histone complexes and histone-associated PTMs as a promising method for discrimination, monitoring, and treatment guiding of cancers with solid organ origin is shown in Table 4.

### 4.1. Cancer Detection

There is growing evidence that high nucleosome levels in the bloodstream are found among cancer patients, especially in advanced stages, which is not observed in healthy individuals [88,118,124,125,126,127,128]. Such nucleosome level increase was most notably observed in lung cancer patients and, to the lowest extent, in prostate cancer patients. Importantly, high levels of circulating nucleosomes were also detected in benign conditions, suggesting low diagnostics power [118]. Similarly, solely measuring the total level of nucleosomes in serum showed a weak ability to differentiate CRC [129] from non-cancerous conditions. However, combining markers of epigenetically modified nucleosomes achieved high sensitivity and specificity of early-stage CRC detection [84,91]. Similarly, distinguishing stage II pancreatic cancer patients from healthy controls and benign disease through five histone-defined biomarkers detected in serum achieved a better prediction score, sensitivity, and specificity, compared to the common pancreatic tumor biomarker, carbohydrate antigen 19-9 (CA 19-9). Interestingly, out of the five marks were the histone variants H2AZ and mH2A1.1 [85], indicating that circulating histone variants could be attractive cancer biomarker candidates. Histone analysis by chromatin immunoprecipitation in the serum of colorectal, pancreatic, breast, and lung cancer patients revealed elevated levels of H3K9me3 and H4K20me3 in all cancer types compared to healthy individuals. Importantly, upon normalization of H3K9me3 and H4K20me3 levels to total nucleosome content, H3K9me3 and H4K20me3 were lower in CRC while remaining elevated in breast cancer compared to healthy controls. Comparing the two histone marks, H4K20me3 was found to discriminate cancer patients from healthy individuals when normalized to nucleosome value in patient serum, while total non-normalized H3K9me3 was able to distinguish colorectal cancer from non-cancerous gastrointestinal diseases [130]. Of note, ELISA-mediated detection of histone marks showed similar values for total H3K9me3 and decreased levels of total H4K20me3 and H3K27me3 in CRC patients compared to healthy individuals [82]. These findings suggest circulating histones as a valuable prognostic marker. However, it is evident that the results are influenced by both the detection method and the data analysis, indicating the need for appropriate standardization.

Recently, Vanderstichele et al. showed that the fragmentation of nucleosome-associated circulating plasma DNA predicted the presence of malignant tumors in 271 plasma samples from patients with an adnexal mass [101]. Of note, nucleosomal DNA fragmentation performed better at distinguishing ovarian cancer malignancies with low chromosomal instability than low-coverage whole genome sequencing [131]. The study suggests circulating plasma nucleosome-DNA complexes could serve as a complementary cancer detection approach, especially in subtypes with a low mutational burden. Similar findings were reported by Cristiano et al. for 236 patients diagnosed with breast, colorectal, lung, ovarian, pancreatic, gastric, or bile duct cancer [132]. Nucleosomal cfDNA fragmentation analysis achieved high sensitivity from 57% to more than 99% at 98% specificity among the analyzed cancer types. However, the model tested whether nucleosome positioning could distinguish cancer patients from healthy individuals, regardless of the cancer type [132]. It would be interesting to address whether the method could discriminate cancers with distinct origins, which could render circulating nucleosomes and nucleosome positioning a powerful tool in cancer care.

**Table 4:** Circulating histones and histones complexes in solid cancers.

Malignancy	Target for Detection	Detection Method	Level/Value	Suggested Function	Reference
Breast	Anti-histones + anti-DNA antibodies	ELISA	High	Detection	[124]
Lung, colorectal, and other gastrointestinal cancers;breast, ovarian, and other gynecological cancers;lymphoma, renal, prostate, and other non-defined cancers	Anti-histones + anti-DNA antibodies	ELISA	High	Detection	[118]
Small-cell lung cancer Head and neck cancer	Anti-histones + anti-DNA antibodies	ELISA	High + consistent decrease	Chemotherapy response	[118]
Pancreatic cancer	Anti-histones + anti-DNA antibodies	ELISA	Fluctuation (high-low-high)	Disease progression following chemotherapy	[118]
CRC	Anti-histones + anti-DNA antibodies	ELISA	High + consistent decrease	Radiochemotherapy response	[125]
Breast	Anti-histones + anti-DNA antibodies	ELISA	High/low	Response prediction to neoadjuvant chemotherapy	[126]
Breast	Anti-histones + anti-DNA antibodies	ELISA	High	Disease progression	[127]
Advanced malignancies	Anti-Citrullinated histone H3	Flow-cytometry	High/low	Prognostic marker	[88]
CRC	H2AK119Ub, H3K9Ac, H3K27Ac, and the global level of nucleosomes	ELISA	High Score-the 4 marks combined	Detection	[84]
Pancreatic stage II	5MC, H2AZ, H3K4Me2, H2AK119Ub and mH2A1.1	ELISA	High Score-the 5 marks combined	Detection	[85]
Colorectal, pancreatic, breast, and lung cancer patients	H3K9me3 and H4K20me3	Chromatin- immunoprecipitation	High total levels	Detection H4K20me3-Cancer vs. healthy H4K20me3-CRC vs. benign gastrointestinal diseases	[130]
CRC	H3K9me3 and H4K20me3	Chromatin- immunoprecipitation	Low, when normalized to total nucleosome content	Detection	[130]
Breast	H3K9me3 and H4K20me3	Chromatin- immunoprecipitation	High, when normalized to total nucleosome content	Detection	[130]
CRC	H4K20me3	ELISA	Low	Detection	[82]
CRC	H3K27me3	ELISA	Low	Detection	[82]
Ovarian	Nucleosomal DNA fragmentation pattern	WGS sequencing and bioinformatics	NA	Detection, specifically cancers with low chromosomal instability	[131]
Breast, colorectal, lung, ovarian, pancreatic, gastric, bile duct	Nucleosomal DNA fragmentation pattern	Genome-wide cell-free DNA fragmentation	NA	Detection	[132]
Hepatocellular carcinoma (HCC)	H3, the canonical H3.1 variant, H3K27me3 and H3K36me3	ELISA	H3K27me3/H3K36me3 ratio-High in disease progression H3K27me3, H3K36me3-low at best therapy response	Responde to sorafenib; Monitoring and disease progression	[133]
Prostate	H3K27me3	ELISA	Very low in metastatic disease	Stage differentiation	[134]
Lung, colorectal, and other gastrointestinal cancers; breast, ovarian, and other gynecological cancers; lymphoma, renal, prostate, and other non-defined, cervical, and pancreatic cancers	Anti-histone and anti-DNA	ELISA	High and further increase	Disease progression; therapy response	[118,125,135,136,137,138,139,140,141]
HCC	Anti-histones + anti-DNA antibodies	ELISA	High; Increased	Radiofrequency ablation (RFA) therapy response	[142]
Non-small-cell lung cancer	Nucleosomal DNA fragmentation pattern	WGS sequencing and bioinformatics	Changes in epigenetic profile	anti-EGFR, anti-ERBB2 response monitoring	[132]
Breast	Nucleosomal DNA fragmentation pattern	WGS sequencing and bioinformatics	Changes in epigenetic profile	Estrogen receptor subtyping	[143]
Breast	Anti-histone and anti-DNA	ELISA	High	Chemotherapy response; Disease progression	[126]
Not specified	5mC and H3K9Me3	ELISA	Low	Detection	[144]
CRC	Nucleosome antibody and antibody against 5mC	ELISA	Low	Detection	[79]
CRC Pancreas, lung, and breast	H3K27me3, H3K9me3, H3K9ac, H3K4me3, H3K36me3, H3K4me1, and H3K27me3	Single-molecule imaging	High; Decrease in some combinatorial patterns (e.g., H3K9me3- and H3K36me3	Detection	[92]
CRC	Anti-histone H3.1 antibody; H3K27Me1, -Me2, -Me3; H3K36Me1, -Me2, -Me3; H3K56Me2 H3K27Ac, H4K20Me1, Me2; H4K4; 17_2Ac, -3Ac, -4Ac; and H2A1R3Cit; H3, H4, H2A1	Nucleosome immunoprecipitation; LC-MS/MS	High	Detection	[91]
Variety of advanced malignancies	Citrullinated histone H3 (H3Cit); Anti-histone H3 and anti-H3Cit antibodies	ELISA	High	Detection	[88]
Cervical	Anti-histone and anti-DNA	ELISA	High + decrease	Chemotherapy response	[136]

### 4.2. Treatment Guidance, Disease, and Therapy Response Monitoring

A limitation of measuring the total level of circulating nucleosomes as a cancer detection approach is the lack of specificity, as various cancer types have shown a high concentration of circulating nucleosomes in retrospective studies. Therefore, simply measuring nucleosomes in the blood lacks the ability to differentiate the primary origin or secondary metastases. However, that provides an opportunity to uncover minimal residual disease and treatment response in an easy-to-use and cost-effective manner. To ensure patients receive the most promising therapy, clinicians require parameters for patient stratification and prediction. Recently, the detection of two PTMs on H3 histone was shown to predict response to the kinase inhibitor sorafenib in hepatocellular carcinoma (HCC) patients. Specifically, increased H3K27me3/H3K36me3 ratio levels in plasma were associated with non-response to sorafenib and disease progression. Conversely, H3K27me3 and H3K36me3 levels were reduced in patients showing the best therapy response compared to baseline levels [133]. Low plasma H3K27me3 levels, but not general nucleosome levels, were also shown to distinguish metastatic prostate cancer from localized/locally advanced disease [134].

Increasing evidence suggests that circulating nucleosomes could be used in therapy response and disease monitoring in various solid cancer types. A transient increase in circulating nucleosome levels (6h and 24h post-treatment), followed by a consistent decrease, indicated positive chemotherapy and radiotherapy responses and remission. In contrast, nucleosome levels remained elevated or continued to rise in non-responder patients and were associated with disease progression [118,125,135,136,137,138,139,140,141]. In line with these findings, Gu et al. found that Radiofrequency ablation (RFA), which is often applied as first-line treatment in HCC patients, causes an increase in circulating histones within 24 h post-therapy [142]. Furthermore, Vanderstichele et al. showed that changes in nucleosome positioning footprint were able to detect anti-EGFR and anti-ERBB2 therapy responsiveness in non-small-cell lung cancer, as it closely reflected expression levels of EGFR or ERBB2 mutant alleles [132]. Similarly, Doebley et al. showed that nucleosome protection profiling could be applied for estrogen receptor subtyping in breast cancer [143]. Together, these findings strongly suggest that circulating histones and histone complexes hold great promise in treatment guidance and cancer monitoring.

### 4.3. Role in Disease Progression

As discussed, changes in circulating histones and histone complexes are prevalent among solid cancers and are associated with disease progression. However, whether and how such markers affect the growth and survival of cancer cells have not been researched elaborately.

Studies have shown the ability of the tumor environment to recruit neutrophils, which could further alter the microenvironment and stimulate tumor progression [145,146,147,148]. Szczerba et al. identified neutrophil-associated CTCs in breast cancer patients and murine models that displayed distinct transcriptomic compared to CTCs alone. Interestingly, differences in gene expression were most notably observed in metastasis-related cell cycle progression pathways, cell-cell junctions, and cytokine-receptor [149]. That brings into question the mechanisms utilized by neutrophils to alter CTC function.

Research by Lorenzo Ferri’s group suggested that extracellular traps facilitate the survival of circulating tumor cells, resulting in metastatic disease progression [150,151]. Utilizing in vivo mouse models and in vitro systems, the authors found that neutrophils are actively participating in metastasis initiation of H59 Lewis lung carcinoma cells and B16-F10 melanoma cells by inducing cell adhesion [150,151]. Metastatic initiation was diminished upon administration of DNAse 1 or neutrophil elastase inhibitor (NEi), directly linking NETs with disease progression. Of note, no histone inhibitors were used in the study [151]. It is plausible that histones are indirectly linked to tumor cell survival and metastasis. However, histones are a crucial component of NETs. Furthermore, real-time analysis via intravital microscopy imaging (IVM) showed tumor cell migration to histone-dense areas. Inhibitors of circulating histones could be used to elucidate the direct role of histones in cancer progression. The small polyanion methyl β-cellobioside per-O-sulfate (mCBS) specifically blocked histones while maintaining NETs integrity [152]. Recently, Wilson et al. showed that NETs could specifically induce the differentiation of IL-17-producing TH17 cells via histone recruitment to Toll-like receptor 2 (TLR2) on naïve T cells and downstream activation of STAT3. Following differentiation, TH17 cells cause further neutrophil activation, creating a positive feedback loop [153]. Given that Th17 T cells are associated with cancer progression [154,155,156], we could hypothesize that histones exert a direct effect on tumorigenesis.

## 5. Market Size and Private Investments

According to the new market research report “Liquid Biopsy Market by Product, Circulating Biomarkers, Technology, Application, End User—Global Forecast to 2026”, the global liquid biopsy market is projected to reach USD 5.8 billion by 2026 from USD 2.5 billion in 2021, at a compound annual growth rate (CAGR) of 18.1%, during the forecast period. The liquid biopsy market is driven by the rising incidence and prevalence of cancer and the increasing preference for non-invasive treatment procedures [157]. Similarly, according to the new market research report “Epigenetics Market by Product & Service, End User—Global Forecast to 2027”, the Epigenetics Market is valued at USD 1.7 billion in 2022 and is expected to reach USD 3.9 billion by 2027 at a CAGR of 18.1% during the forecast period [158].

Several companies, such as VolitionRx or EpiGentek, currently develop circulating histones-based platforms to help diagnose and monitor certain cancers and diseases associated with the release of histones or nucleosomes in the bloodstream. In summary, the clinical development of liquid biopsies for cancer, a revolutionary screening tool, can be looked at with great optimism.

## 6. Conclusions

In conclusion, measuring histones and histone complexes levels and modifications has achieved high sensitivity and specificity in detecting and monitoring various cancers, providing an exciting direction for non-invasive cancer diagnostics. The high stability and abundance of circulating histones, compared to cfDNA and CTCs, respectively, support the application of histone-based liquid biopsies in cancer diagnostics. The methods of histone detection in circulation are continuously advancing, achieving fast and robust results at a low cost. Nevertheless, standardization of the approach and the analysis is needed to define the potential of histones and nucleosomes as cancer biomarkers.

Research has been focused on comparing cancer patients to healthy individuals and patients with benign diseases. While an increasing number of studies are highlighting circulating histones as markers and predictors of hematologic malignancies and solid cancer, studies on the ability of circulating histones to distinguish individual cancer types with distinct origins are currently lacking and should be envisaged in the near future.

## Figures and Tables

**Table 1:** Characteristics of the different types of liquid biopsies.

Type of Liquid Biopsy	Stability	Detection Method	Specificity	Sensitivity
Circulating tumor DNA	*	PCR, sequencing (various types); Nanomaterials-based ctDNA analysis; Electrochemical ctDNA detection; Surface-enhanced Raman scattering (SERS)	**	****
Circulating tumor cells	****	Flow-cytometry; nano/micro magnetic particles; Microfluidics; mechanical filtration; Hydrodynamics; electrokinetics; acoustophoresis	***	**
Tumor extracellular vesicles	**	Centrifugation, density gradient; chromatography; fractionation immunoaffinity; lipidomics/mass spectrometry; flow cytometry; microfluidics; lateral-flow immunoassay (LFIA); nanoparticle tracking analysis (NTA)	***	***
Circulating histones	***	ELISA, proteomics, Flow-cytometry	***	****

*—low; **—low to moderate; ***—moderate to high; ****—high.

**Table 2:** Characterization of ELISA, Proteomics, and ImageStreamX for the analysis of circulating histones and histone complexes. The advantages and disadvantages of the indicated parameters are illustrated through an asterisk-based system.

Detection Method	Sensitivity	Multiplex	Bias	Easy to Use	Easy to Access	Expensiveness	Speed
ELISA	****	**	****	****	***	**/***	**
Proteomics	****	****	*	*	*	*	**
ImageStream	****	***	***	**/***	*	**/***	****

*—low; **—low to moderate; ***—moderate to high; ****—high.

**Table 3:** Circulating histones and histones complexes in hematologic malignancies.

Malignancy	Target for Detection	Detection Method	Level	Suggested Function	Reference
Leukemia	Histone—dsDNA complex	ELISA	High	Disease progression Chemotherapy resistance	[115,116]
Leukemia	NA	NA	Injection of 200 µg/mL H1 histone	Cytotoxicity of tumor cells	[117]
Burkitt’s lymphoma	NA	NA	Injection of 200 µg/mL H1 histone	Inhibits tumor growth	[117]
Lymphoma	Anti-histone, anti-DNA-antibodies	ELISA	High	Detection	[118]
Non-Hodgkin lymphoma (NHL)	Nu.Q-H3.1	ELISA	High	Detection	[119]
Acute myeloid leukemia (AML)	Nu.Q-H3.1	ELISA	High	Detection	[119]
Acute lymphocytic leukemia (ALL)	Nu.Q-H3.1	ELISA	High	Detection	[119]
AML	Anti-histone, anti-DNA-antibodies	ELISA	Initial rise + Decrease following treatment	Chemotherapy response	[121]
Lymphoma	UV intensities of individual DNA fragments	Detection on 2% agarose gel	High	Detection of disease progression	[122]

## Data Availability

Not applicable.
